# H/ACA Small Ribonucleoproteins: Structural and Functional Comparison Between Archaea and Eukaryotes

**DOI:** 10.3389/fmicb.2021.654370

**Published:** 2021-03-11

**Authors:** Dominic P. Czekay, Ute Kothe

**Affiliations:** Department of Chemistry and Biochemistry, Alberta RNA Research and Training Institute, University of Lethbridge, Lethbridge, AB, Canada

**Keywords:** H/ACA RNA, pseudouridine, RNA modification, ribosome biogenesis, pre-rRNA processing, telomerase, Dyskeratosis congenita, dyskerin

## Abstract

During ribosome synthesis, ribosomal RNA is modified through the formation of many pseudouridines and methylations which contribute to ribosome function across all domains of life. In archaea and eukaryotes, pseudouridylation of rRNA is catalyzed by H/ACA small ribonucleoproteins (sRNPs) utilizing different H/ACA guide RNAs to identify target uridines for modification. H/ACA sRNPs are conserved in archaea and eukaryotes, as they share a common general architecture and function, but there are also several notable differences between archaeal and eukaryotic H/ACA sRNPs. Due to the higher protein stability in archaea, we have more information on the structure of archaeal H/ACA sRNPs compared to eukaryotic counterparts. However, based on the long history of yeast genetic and other cellular studies, the biological role of H/ACA sRNPs during ribosome biogenesis is better understood in eukaryotes than archaea. Therefore, this review provides an overview of the current knowledge on H/ACA sRNPs from archaea, in particular their structure and function, and relates it to our understanding of the roles of eukaryotic H/ACA sRNP during eukaryotic ribosome synthesis and beyond. Based on this comparison of our current insights into archaeal and eukaryotic H/ACA sRNPs, we discuss what role archaeal H/ACA sRNPs may play in the formation of ribosomes.

## Introduction

Ribosomes are macromolecular components present in all living cells responsible for protein biosynthesis, one of the energetically most expensive processes in cells. Ribosome biogenesis begins with the transcription of ribosomal RNA (rRNA), which in both archaea and eukaryotes is mostly transcribed as a long precursor containing individual segments of rRNA although some archaea also have separate rRNA genes (Yip et al., 2013). During the early stages of ribosome biogenesis, the nascent pre-rRNA is subject to many site-specific RNA modifications, the most abundant of which are 2′-O-methylations and pseudouridines (Maden, 1990; Kos and Tollervey, 2010; Yip et al., 2013).

**FIGURE 1:**
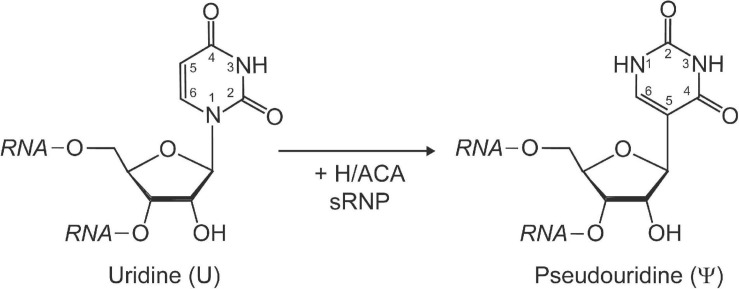
Schematic representation of the isomerization of a uridine to pseudouridine by H/ACA sRNPs. Pseudouridine is characterized by a unique C–C glycosidic bond linking C1′ of the ribose with C5 of the base as well as an extra imino group (N1) with hydrogen bonding potential within the base. The Watson-Crick face in pseudouridine is unchanged and allows base-pairing with adenine.

Pseudouridine is a structural isomer of uridine initially discovered using two-dimensional paper chromatography of yeast RNA extracts (Davis and Allen, 1957). This RNA modification is characterized by its unique C–C glycosidic bond (Figure 1). The isomerization of uridine to pseudouridine results in an additional imino group acting as a hydrogen bond donor on the Hoogsteen edge of the base. Pseudouridine has been demonstrated to be more thermodynamically favorable than uridine when present in short duplexes of RNA (Davis, 1995; Kierzek et al., 2014). This can be partially explained by the fact that in crystal structures pseudouridine is observed to coordinate a water molecule between its nucleobase and nearby sugar-phosphate backbone, providing a rigidifying effect to the local RNA fold and increasing base stacking interactions (Arnez and Steitz, 1994).

The formation of pseudouridine is catalyzed by a conserved class of enzymes known as pseudouridine synthases. In bacteria, these enzymes exist solely as stand-alone proteins, which both recognize the modification site in rRNA or tRNA and catalyze their modification (reviewed in Hamma and Ferre-D’Amare, 2006). While archaea and eukaryotes also contain stand-alone enzymes for this purpose (reviewed in Rintala-Dempsey and Kothe, 2017), a more sophisticated system employing H/ACA sRNPs is responsible for nearly all rRNA modifications (Ganot et al., 1997a; Ni et al., 1997; Yu and Meier, 2014). H/ACA sRNPs are named after the H/ACA guide RNA component that determines their sequence specificity. In 1997, two groups discovered the hitherto unknown function of H/ACA RNAs and their associated proteins in directing the site-specific pseudouridylation in rRNA triggering a plethora of studies in yeast and other eukaryotes that provides the basis for our current understanding of H/ACA sRNP function (Ganot et al., 1997a; Ni et al., 1997). Eukaryotic H/ACA sRNPs are further distinguished as H/ACA small nucleolar RNPs (snoRNPs) or H/ACA small Cajal-body-specific RNPs (scaRNPs), which localize and function in nucleoli and Cajal bodies, respectively (Darzacq et al., 2002). Shortly after the description of H/ACA sRNAs in eukaryotes, their presence was also verified in archaea (Watanabe and Gray, 2000; Tang et al., 2002). Considering the lack of subnuclear compartments in archaea, the archaeal counterparts are simply designated as H/ACA snoRNP-like, or more commonly as H/ACA small ribonucleoproteins (sRNPs) (Omer et al., 2003). In this review, we will explore and compare the structures of archaeal and eukaryotic H/ACA sRNPs, the variety of functions of H/ACA sRNPs, and discuss what is known about their assembly and implications on ribosome biogenesis and beyond.

## H/ACA sRNPs Share a Common Structural Core Organization

**FIGURE 2:**
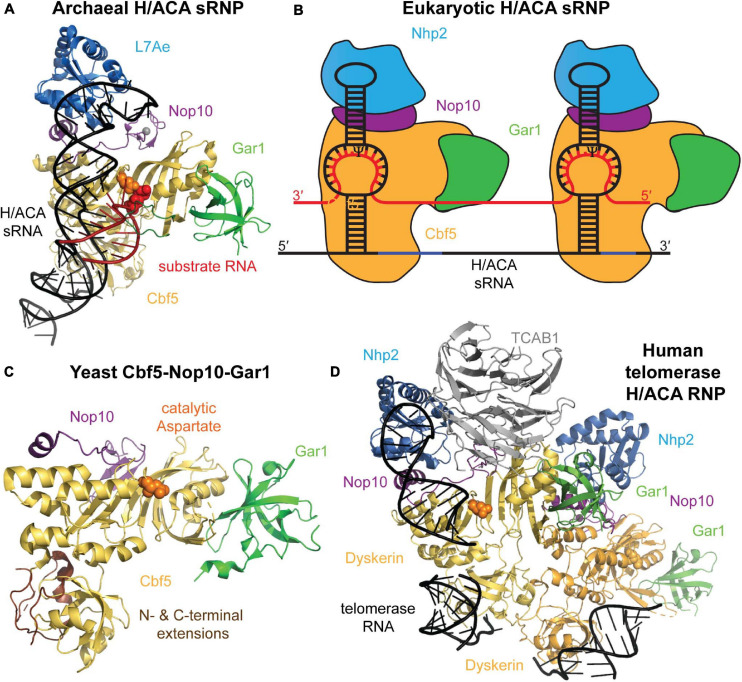
Archaeal and eukaryotic H/ACA sRNP structure. **(A)** Crystal structure of an H/ACA sRNP bound to substrate RNA (red) from *P. furiosus* (PDB ID: 3HAY) (Duan et al., 2009). The single-hairpin H/ACA sRNA (black) binds to the four H/ACA proteins: Cbf5 (orange), Nop10 (purple), L7Ae (light blue), and Gar1 (green). In the active site of Cbf5, the catalytic aspartate residue is depicted in orange sphere adjacent to the target uridine (red sticks). **(B)** Schematic representation of the typical two-hairpin structure of a eukaryotic H/ACA sRNP bound to a target RNA (red). Each hairpin of the H/ACA snoRNA (black) is assumed to bind a complete set of H/ACA proteins. The proteins and RNAs are colored as in **(A)**. Note that Nhp2 is the eukaryotic homolog of archaeal L7Ae. **(C)** Crystal structure of the yeast Cbf5-Nop10-Gar1 complex (PDB ID: 3U28) shown in a similar conformation as the archaeal H/ACA sRNP complex in **(A)** (Li et al., 2011b). In eukaryotes, the PUA of Cbf5 (bottom) is larger than in archaea due to N- and C-terminal extensions shown in brown. **(D)** Structural model of the H/ACA sRNP complex assembled on the 3′ end of human telomerase RNA based on a cryo-electron microscopy structure of human telomerase (Nguyen et al., 2018). Two sets of all H/ACA proteins (including the human homolog of Cbf5 called dyskerin) are observed as well as the Telomerase Cajal body protein 1 (TCBA1, gray). All structure representations were prepared using Pymol software.

A mature H/ACA sRNP particle is composed of four different core proteins that assemble onto a H/ACA guide RNA scaffold (Figure 2). The archaeal proteins and their eukaryotic homologs that constitute H/ACA sRNPs are: L7ae (Nhp2 in eukaryotes), Nop10, Gar1, and the catalytic component, Cbf5 (dyskerin in humans) (Watanabe and Gray, 2000; Rozhdestvensky et al., 2003). Li and Ye (2006) reported the first structure revealing the organization of an archaeal H/ACA sRNP which was followed by a number of further structures of archaeal H/ACA sRNPs including structures showing the recognition of target RNA (Liang et al., 2007a; Duan et al., 2009; Liang B. et al., 2009; Zhou et al., 2010). The overall structural similarity of archaea and eukaryotic H/ACA sRNPs as well as some critical differences have subsequently been revealed by a structure of the *S. cerevisiae* Cbf5-Nop10-Gar1 complex, and more recently, by a cryo-electron microscopy structure of human telomerase, containing a H/ACA sRNP assembled on the 3′ end of human telomerase RNA (Li et al., 2011b; Nguyen et al., 2018). In the following, we will introduce the structural features of the H/ACA sRNP components and discuss their conservation and differences between archaea and eukaryotes.

H/ACA guide RNAs in archaea and eukaryotes have a few notable differences. In most studied eukaryotes (albeit with few exceptions like trypanosomes; Liang et al., 2004), all H/ACA snoRNAs conform to a hairpin-hinge-hairpin secondary structure where each hairpin is followed by one of two conserved consensus sequences, the H box (consensus ANANNA) and the ACA box, respectively. Within the ACA box, the adenines are most conserved, and alternative sequences (AUA, AAA, or AGA) can be found (Ganot et al., 1997b). In all cases, the ACA box is located strictly three nucleotides upstream of the 3′ end of the RNA (Balakin et al., 1996; Ganot et al., 1997b). However, some archaea display also highly atypical H/ACA RNA features (Bernick et al., 2012). Instead of the two-hairpin structure observed in almost all eukaryotes like yeast and humans, the vast majority of archaeal H/ACA sRNAs contain just one hairpin followed by an ACA box, but in rare instances archaeal H/ACA sRNAs have two or three hairpins (Rozhdestvensky et al., 2003). In both eukaryotes and archaea, the H and ACA box elements are necessary for H/ACA sRNP accumulation, localization, and pseudouridylation activities *in vivo* (Balakin et al., 1996; Ganot et al., 1997b; Bortolin et al., 1999; Narayanan et al., 1999; Caton et al., 2018). As obvious from the crystal structure, the single-hairpin H/ACA sRNA in archaea is bound by one set of core proteins (Li and Ye, 2006), and in analogy, it is assumed that each hairpin of H/ACA sRNAs in eukaryotes also binds a complete set of H/ACA proteins which is further supported by the set of two H/ACA proteins observed bound to human telomerase RNA (Figure 2; Nguyen et al., 2018).

Within each hairpin, H/ACA RNAs contain single-stranded pockets, generally known as pseudouridylation pockets. The unpaired nucleotides in the pocket provide pseudouridylation specificity by selecting a uridine in the target RNA whose flanking nucleotides complement those in the H/ACA sRNA (Ganot et al., 1997a). Target RNA binding forms a three-way junction at which the targeted uridine as well as a nucleotide 3′ of the target uridine remain unpaired in the center of the guide RNA pocket, and the target uridine is inserted into the active site of Cbf5 for modification (Liang et al., 2007a; Wu and Feigon, 2007; Liang B. et al., 2009). In eukaryotes, at least 8 base-pairs are required between the H/ACA sRNA and its target RNA with no less than three base-pairs on either side of the pseudouridylation pocket in order to allow for efficient pseudouridine formation (De Zoysa et al., 2018; Kelly et al., 2019). Across all functional H/ACA sRNAs, there is a defined distance between the site of pseudouridylation and the nearest downstream consensus sequence element (H box or ACA box), but this distance varies slightly between eukaryotes and archaea (Tang et al., 2002; Toffano-Nioche et al., 2015). In archaea this distance is typically 14–16 nt, whereas in eukaryotes it is generally 15–16 nt. Functionally, this distance acts as a molecular ruler that ensures proper positioning of the guide RNA relative to the active site in Cbf5 such that a bound substrate RNA target uridine can be positioned for catalysis (Caton et al., 2018).

A notable difference between eukaryotic and archaeal H/ACA sRNAs is the presence of a conserved K-turn or K-loop motif in the upper portion of the hairpin above the pseudouridylation pocket in archaeal H/ACA sRNA which is absent in eukaryotic H/ACA sRNAs (Rozhdestvensky et al., 2003). The K-turn or kink-turn motif is a common RNA motif that results in a characteristic kink in an RNA helix as first observed in ribosomal RNA (Klein et al., 2001). The kink is caused by a three-nucleotide internal bulge that is closed on one site by two canonical G-C base pairs and that is flanked on its other site by two sheared G-A base pairs. Whereas some archaeal H/ACA sRNAs have a longer upper hairpin stem harboring a K-turn motif, other archaeal H/ACA sRNAs with a shorter upper stem contain a variation of this motif called K-loop. Here, the G-A base-pairs are present, but instead of a 3-nucleotide bulge a 7-nucleotide loop is found. Notably, both the K-turn and the K-loop motif are always located 5–6 nucleotides above the pseudouridylation pocket (Rozhdestvensky et al., 2003).

The catalytic core component of H/ACA sRNPs is the protein Cbf5, a pseudouridine synthase of the TruB family. This family is defined by the essential PseudoUridine synthase and Archaeosine transglycosylase (PUA) domain, a common RNA binding domain that contributes to H/ACA sRNA binding in Cbf5 by interacting with the lower stem and the H or ACA box (Hamma et al., 2005; Hamma and Ferre-D’Amare, 2006). One notable difference between eukaryotic and archaeal Cbf5 is the presence of N- and C-terminal extensions in the eukaryotic protein that both contribute to a larger PUA domain but may also be partially unstructured based on the presence of many charged residues (Figure 2). The catalytic domain of Cbf5 harbors the core fold and conserved active site cleft residues that are shared across all pseudouridine synthase families (Hamma et al., 2005; Hamma and Ferre-D’Amare, 2006). The active site is characterized by the presence of a strictly conserved catalytic aspartate residue that is required for nucleophilic attack during isomerization (Figure 2; Huang et al., 1998; Veerareddygari et al., 2016). Additional active site residues include a conserved basic residue and a tyrosine residue that stacks with the target uracil base (Ferre-D’Amare, 2003). In TruB, the bacterial homolog of Cbf5, the conserved basic residues are shown to participate in an electrostatic network important for modification; meanwhile, the conserved tyrosine is suggested to maintain active site structure and may act as a general base during catalysis (Phannachet et al., 2005; Friedt et al., 2014). Interestingly, Cbf5 is an essential gene in eukaryotes, but it can be deleted in *Haloferax volcanii* suggesting a differential importance of H/ACA sRNPs in eukaryotes and archaea (Jiang et al., 1993; Blaby et al., 2011).

The pseudouridine synthase Cbf5 tightly interacts along its catalytic domain with the protein Nop10, a small (<10 kDa) protein that binds Cbf5 independent of other proteins or RNA (Hamma et al., 2005). Nop10 is organized into two domains that are separated by a linker. Although the linker and C-terminal domain of Nop10 are mostly unstructured in solution, Nop10 adopts structure upon binding to Cbf5 (Hamma et al., 2005; Khanna et al., 2006; Reichow and Varani, 2008). When bound, the central region of Nop10 supports the boundaries of the Cbf5 active site, and is speculated to potentially influence active site dynamics (Hamma et al., 2005). Unique to archaeal Nop10 is the presence of a highly stable N-terminal zinc-binding ribbon that is replaced by a smaller, only partially stable, β-hairpin in eukaryotic counterparts (Khanna et al., 2006). When in complex with Cbf5, a pair of solvent-exposed Nop10 aromatic residues moderately contribute to binding of the H/ACA RNA (Hamma et al., 2005). Moreover, Nop10 seems to stabilize the active site of Cbf5 thereby enhancing its catalytic activity (Kamalampeta and Kothe, 2012).

The third H/ACA sRNP protein is Gar1, an essential protein containing one large central domain flanked by two Glycine-Arginine Rich (GAR) regions, which are common amongst other yeast nucleolar proteins (Girard et al., 1992; Bagni and Lapeyre, 1998). Archaeal homologs of Gar1 are substantially smaller in size, as they lack both GAR regions found in their eukaryotic counterparts (Bridger et al., 2012). Consequently, only the central portion of the eukaryotic protein is conserved in archaea. Strikingly, a Gar1 central-domain only variant in yeast was demonstrated to be sufficient in performing all essential functions of full-length Gar1 *in vivo*, rescuing growth and pre-rRNA processing defects observed in Gar1-deficient strains (Girard et al., 1992, 1994). The central domain of Gar1 interacts with the catalytic domain of Cbf5, but is not in direct contact with the H/ACA sRNA (Figure 2; Li and Ye, 2006). Instead, Gar1 also enhances Cbf5’s catalytic activity similar to Nop10 (Kamalampeta and Kothe, 2012), and it is critical for product release (Duan et al., 2009). The later function is achieved through an interaction of Gar1 with the so-called thumb loop of Cbf5: in the substrate-free, open state, Gar1 binds the thumb loop allowing Cbf5 to recruit substrate RNA. Subsequently, Cbf5’s thumb loop is released from Gar1 and binds over the substrate RNA thereby stabilizing it in the active site of Cbf5. In order to allow for product release after pseudouridine formation, Gar1 has to once again bind the thumb loop of Cbf5 to allow for target RNA dissociation (Duan et al., 2009). Interestingly, yeast Gar1 has been reported to directly bind the essential H/ACA snoRNAs snR30 and snR10 (Bagni and Lapeyre, 1998). While this interaction is not observed in the H/ACA sRNP structures reported to-date, it could be that eukaryotic Gar1 fulfills additional functions by directly interacting with RNA.

The upper stem of the H/ACA sRNA is bound by the archaeal protein L7Ae or its respective eukaryotic homolog Nhp2 (Rozhdestvensky et al., 2003). L7Ae is a member a large family of RNA-binding proteins that specifically recognize K-turn and K-loop motifs (Rozhdestvensky et al., 2003; Hamma and Ferre-D’Amare, 2004; Gagnon et al., 2010). Notably, L7Ae is also a core component of archaeal C/D sRNPs where L7Ae also recognizes a K-loop motif. While Nhp2 is the eukaryotic homolog of L7Ae, it has lost the ability to specifically bind K turns or K loops in agreement with the absence of these motifs in eukaryotic H/ACA sRNPs (Henras et al., 2001). Moreover, Nhp2 is restricted to H/ACA sRNPs only whereas eukaryotic C/D sRNPs contain the homolog Snu13p/14k which continues to recognize K turns and loops. Nevertheless, Nhp2 has retained the general ability to bind RNA (Henras et al., 2001). Unlike L7Ae, which shows little affinity for other H/ACA sRNP proteins, Nhp2 tightly binds to Nop10 in eukaryotic RNPs (Hamma et al., 2005). As a result, the recruitment of L7Ae and Nhp2 to the H/ACA sRNP differs: whereas Nhp2 is anchored to the H/ACA RNP through a protein-protein interaction with Nop10, L7Ae relies on binding to the K-turn of the H/ACA guide RNA and only forms a weak binding interface with Nop10 (Wang and Meier, 2004). Presumably, the conserved distance of 5–6 nucleotides between the pseudouridylation pocket and the K-turn or K-loop motif in archaeal H/ACA sRNA is required to allow for these week L7Ae-Nop10 interactions (Rozhdestvensky et al., 2003). Notably, both Nhp2 and L7Ae play an important role in anchoring the top of an H/ACA guide RNA hairpin and to position the pseudouridylation pocket in close proximity of the active site of Cbf5 which is important for pseudouridylation activity (Liang et al., 2007a, 2008; Caton et al., 2018). Thus, Nhp2 and L7Ae differ in their molecular interactions, but seem to fulfill the same function.

## Functional Roles of H/ACA sRNPs in Ribosome Formation and Beyond

**FIGURE 3:**
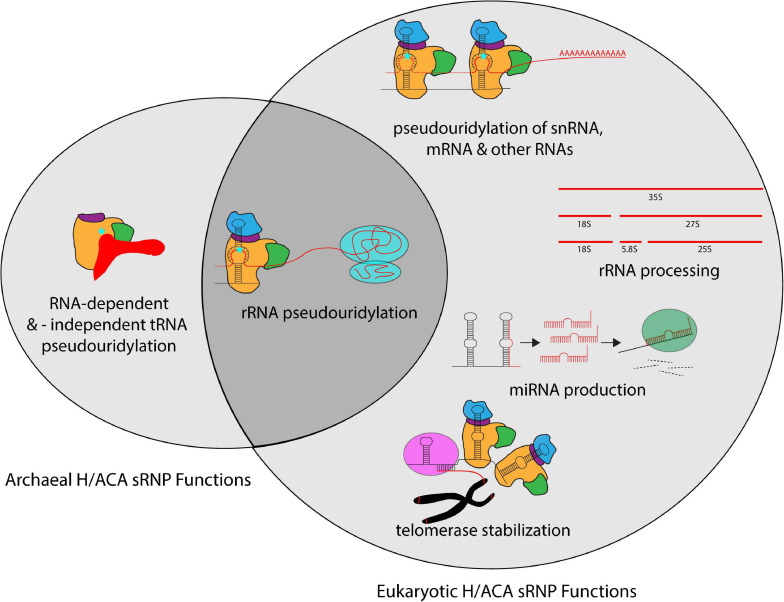
Overview of the different cellular processes that involve H/ACA sRNPs in archaea and eukaryotes. Whereas most known H/ACA sRNPs are responsible for rRNA pseudouridylation, many additional modification targets in snRNAs, mRNAs and other non-coding RNAs have been identified in eukaryotes. In contrast, the archaeal H/ACA proteins are also capable of pseudouridylating tRNA both in a guide RNA-dependent and -independent manner. In addition to the RNA modification activities, eukaryotic H/ACA sRNPs are also implicated in rRNA processing, miRNA production, and telomerase stabilization.

H/ACA sRNPs play roles in several cellular pathways including ribosome biogenesis, but also in many other RNA-related processes (Figure 3). The most well defined and characteristic role of H/ACA RNPs is the site-specific introduction of pseudouridines in rRNA during ribosome synthesis (Bousquet-Antonelli et al., 1997; Ganot et al., 1997a; Ni et al., 1997). While the specific role of individual pseudouridines in rRNA remains unclear, collectively pseudouridines are critical for ribosome function, and the removal of select pseudouridines via the deletion of the respective H/ACA guide RNAs causes changes in ribosome structure and function (Penzo and Montanaro, 2018). Importantly, pseudouridines occur with the greatest frequency in functionally important regions of the ribosome such as the peptidyl transferase center, the decoding center, and intersubunit bridges (Bakin et al., 1994; Liang et al., 2007c). In yeast, the removal of H/ACA guide RNAs introducing pseudouridines in these regions influences ribosome structure, translation rate, translational fidelity, and biogenesis (King et al., 2003; Baxter-Roshek et al., 2007; Decatur et al., 2007; Baudin-Baillieu et al., 2009; Liang X.H. et al., 2009; Polikanov et al., 2015; Sloan et al., 2017). However, since archaea are less amenable to genetic manipulation, our understanding of the exact roles of rRNA pseudouridylation for the archaeal ribosome is lagging. Interestingly, for the organisms studied so far, it seems that archaeal ribosomes contain a much lower number of pseudouridines (e.g., 5 in *Sulfolobus acidocaldarius*) compared to their eukaryotic counterparts and even compared to some bacteria like *Escherichia coli* with 11 rRNA pseudouridines (Massenet et al., 1999; Hamma and Ferre-D’Amare, 2006). However, the pseudouridines detected in archaeal rRNA also reside in critical regions, namely the peptidyltransferase center and helix 69 of the 23S rRNA, and similar positions are also modified in bacteria (Ofengand and Bakin, 1997; Massenet et al., 1999; Blaby et al., 2011). Based on the conservation of rRNA pseudouridylation in all kingdoms of life, it seems therefore reasonable to assume that rRNA modification by archaeal H/ACA sRNPs plays in general similar roles in ribosome synthesis and translation as in eukaryotes.

The ribosomal A-site acts as the binding site for incoming aminoacyl-tRNAs during protein synthesis and contains several pseudouridines in eukaryotes. Removal of pseudouridines within the yeast ribosomal A-site alters the structure of the A-site, changing the positioning of critical bases involved in tRNA accommodation (Baxter-Roshek et al., 2007). The yeast A-site finger contains four pseudouridines that cause slight increases in frameshifting when removed individually; however, the removal of all four pseudouridines causes elevated UGA stop codon readthrough with increased + 1 frameshifting (Baudin-Baillieu et al., 2009). Deleting pseudouridines together with 2′-O-methylations in the ribosomal A-site further affects translation fidelity (Baudin-Baillieu et al., 2009). Given the sparsity of pseudouridines in archaeal rRNA, it is currently not clear whether some pseudouridines are fulfilling similar roles in the archaeal A site. Helix 69 of 25S rRNA is an important region of the ribosome and is part of the intersubunit bridge connecting the small and large ribosomal subunit. Depletion of rRNA modifications in this intersubunit bridge (Helix 69), which includes four pseudouridine residues, results in decreased growth rate, increased antibiotic sensitivity, and increased frameshifting during translation in yeast (Liang et al., 2007c). Most likely, the conserved pseudouridines in archaeal helix 69 have a similar function.

Aside from ribosomal RNA, pseudouridines have also been discovered in tRNA, small nuclear RNA (snRNA), long non-coding RNA (lncRNA) and mRNA in eukaryotes (Carlile et al., 2014; Lovejoy et al., 2014; Schwartz et al., 2014; Li et al., 2015). Many (but not all) pseudouridines in snRNA are introduced by H/ACA RNAs called small Cajal body RNAs (scaRNAs), a subset of H/ACA sRNAs that do not exist in archaea. In addition to the H and ACA boxes common to all H/ACA RNAs, box H/ACA scaRNAs contain one additional sequence element named the CAB box (consensus UGAG) that is located at the terminal loops of each hairpin (Richard et al., 2003). Moreover, several pseudouridines in yeast and human mRNAs are dependent on Cbf5/dyskerin and are therefore most likely introduced by H/ACA sRNPs (Carlile et al., 2014; Schwartz et al., 2014; Li et al., 2015). In archaea, it is currently unknown whether H/ACA sRNPs can also target RNAs other than rRNA, but at least computational predictions suggest that this possibility should not be ruled out (Toffano-Nioche et al., 2015).

Transfer RNA (tRNA) is one of the most highly modified RNAs within all cells. Notably, the pseudouridylation of position 55 at the TΨC arm, is universally conserved across all domains of life in all elongator tRNAs. In eukaryotes, this pseudouridine is introduced by the standalone pseudouridine synthase Pus4, but interestingly in archaea, Ψ55 can be introduced by both the standalone enzyme Pus10 (which is not related to Pus4) as well as by Cbf5 (Roovers et al., 2006; Gurha and Gupta, 2008). Strikingly, in the latter scenario, Cbf5 is capable of introducing the pseudouridine at position 55 in an RNA-independent manner, i.e., without H/ACA sRNA, and this activity is enhanced by the presence of the Nop10 and Gar1 proteins (Roovers et al., 2006; Kamalampeta and Kothe, 2012; Fujikane et al., 2018). To bind the tRNA in the absence of H/ACA sRNA, the archaeal Cbf5 PUA domain binds the CCA 3′ end of the tRNA tightly highlighting the versatility of the PUA domain in either binding the ACA motif of H/ACA sRNAs or the CCA motif of tRNAs (Roovers et al., 2006). However, Cbf5 is non-essential in *H. volcanii* in contrast to Pus10 indicating that *in vivo* Pus10 is the predominant tRNA Ψ55 modification enzyme (Blaby et al., 2011). In addition to this RNA-independent modification of tRNAs by archaeal Cbf5, it has also been reported that at least in some archaeal species such as *Sulfolobus solfataricus* the pseudouridine in position 35 of pre-tRNA^Tyr^ can be generated in an RNA-dependent mechanism by a complete H/ACA sRNP (Muller et al., 2009).

Whereas pseudouridylation by H/ACA sRNPs is their most studied activity, it is presumably not their most important cellular function. Notably, as mentioned, the catalytic protein of H/ACA sRNP, Cbf5, is not essential in archaea suggesting that ribosome biogenesis can occur in the absence of pseudouridylation in archaea (Blaby et al., 2011). Interestingly, the same is true in yeast. Whereas Cbf5 is essential (Jiang et al., 1993), yeast strains expressing only catalytically inactive Cbf5 show a significant growth defect, but are viable (Zebarjadian et al., 1999). These observations raise the intriguing question regarding additional functions of H/ACA sRNPs beyond RNA modification which have been identified in eukaryotes, but not (yet) in archaea (Mitchell et al., 1999a; Vos and Kothe, 2020).

Interestingly, modification H/ACA sRNAs are usually non-essential in eukaryotes, but this is not true for all H/ACA sRNAs providing insight into the most critical cellular function of H/ACA sRNPs. The one essential eukaryotic H/ACA sRNA is *S. cerevisiae* snR30/human U17 (Bally et al., 1988). Notably, there is no identified homologs of the snR30 RNA in archaea. Unlike typical H/ACA sRNAs, snR30/U17 has no known sites of pseudouridylation but instead has a crucial role for the processing of 35S pre-rRNA to generate 18S rRNA (Vos and Kothe, 2020). Together with the core H/ACA proteins and several other ribosome biogenesis factors, snR30 facilitates the early endo-nucleolytic 35S pre-rRNA cleavage events (Zebarjadian et al., 1999; Atzorn et al., 2004). Therein, snR30 also base-pairs with rRNA in an unpaired pocket of its 3′ hairpin; however this interaction resides at the bottom rather than the top of the pocket and thus differs significantly from the rRNA interactions of modification H/ACA sRNPs (Fayet-Lebaron et al., 2009). The detailed molecular mechanism and the architecture of the snR30 H/ACA sRNP remain unknown, but evidently this complex is responsible for the most important function of eukaryotic H/ACA sRNPs during ribosome assembly. Although not essential, the yeast snR10 H/ACA sRNP is similarly implicated in 35S pre-rRNA processing, and consequently its deletion also increases cell doubling time, results in accumulation of 35S pre-rRNA, and causes a cold-sensitive phenotype (Tollervey, 1987; King et al., 2003). Given that processing of archaeal rRNA occurs entirely differently using an archaeal-specific splicing mechanism (summarized in Yip et al., 2013; Clouet-d’Orval et al., 2018), it seems unlikely that an archaeal H/ACA sRNP fulfills a similar role during rRNA processing as the eukaryotic snR30/U17 H/ACA sRNP, but it cannot be excluded that H/ACA sRNPs are differently involved in archaeal ribosome formation.

One interesting function of H/ACA sRNAs observed exclusively in vertebrates is the stabilization of telomerase RNA. The 3′ end of vertebrate telomerase RNA folds into a secondary structure that strongly resembles an H/ACA sRNA, and accordingly the 3′ end of telomerase RNA assembles with two complete sets of box H/ACA core proteins (Figure 2D; Mitchell et al., 1999a; Chen et al., 2000; Dragon et al., 2000). Similar to H/ACA sRNAs that direct pseudouridylation, telomerase RNA contains consensus H and ACA sequences that are also essential for its accumulation, 3′ end processing, and telomerase activity (Mitchell et al., 1999a). This function of vertebrate H/ACA sRNPs has been strongly implicated with a human premature aging syndrome called Dyskeratosis congenita characterized by leukoplakia, nail dystrophy, bone marrow failure, and increased susceptibility to some forms of cancer (Dokal, 2000). The disease has three forms: autosomal dominant, autosomal recessive, and X-linked (X-DC), which is the most severe of all forms. Many X-DC patients have mutations in dyskerin, the human homolog of *Cbf5* (Heiss et al., 1998) which cluster in the PUA domain as well as N- and C-terminal extensions of dyskerin which envelop the PUA domain (Hamma et al., 2005; Rashid et al., 2006). Notably, the mutated residues are generally conserved in eukaryotic Cbf5/dyskerin, but not in its archaeal homolog. In accordance with the role of the PUA domain for the binding to the ACA box in H/ACA sRNAs, many X-DC dyskerin variants do not bind telomerase RNA leading to its destabilization (Ashbridge et al., 2009). As a consequence, one key symptom of X-DC is the shortening of telomeres in cells derived from X-DC patients as well as reduced telomerase activity in primary cells (Mitchell et al., 1999b; Vulliamy T.J. et al., 2001). In addition, it was shown for certain X-DC mutations that they also impair rRNA pseudouridylation and reduce rRNA processing (Mochizuki et al., 2004), and in a mouse model with reduced dyskerin expression, which recapitulates Dyskeratosis congenita features, ribosomal defects appear before telomere shortening (Ruggero et al., 2003). The less severe autosomal dominant form of Dyskeratosis congenita is characterized by mutations that remove a portion of the H/ACA RNA-like structure of telomerase RNA (Vulliamy T. et al., 2001). Unlike dyskerin mutations in X-DC, autosomal dominant mutations do not reduce binding of dyskerin to telomerase RNA (Ashbridge et al., 2009). Another autosomal recessive form of Dyskeratosis congenita is also linked to H/ACA RNPs and is caused by mutations in the *nop10* or *nhp2* genes (Walne et al., 2007; Vulliamy et al., 2008). In summary, the importance of human H/ACA sRNPs for telomere maintenance and ribosome biogenesis is underlined by the molecular defects observed in the different forms of Dyskeratosis congenita.

Lastly, the functions of eukaryotic H/ACA sRNPs extend even further beyond RNA modifications, telomerase stabilization, and rRNA processing (McMahon et al., 2015). In at least one instance, a human H/ACA RNA has been shown to function as a micro RNA (miRNA) after processing by the Dicer enzyme *in vivo* (Ender et al., 2008). Many small RNAs (20–26 nt in length) created from ACA45, normally responsible for directing pseudouridylation of U37 in U2 spliceosomal RNA (snRNA), can stably associate with Argonaute (Ago) proteins and direct the degradation of transcriptional regulator CDC2L6 mRNA (Ender et al., 2008). Notably, other human miRNAs might also be derived from H/ACA sRNA-like precursors (Scott et al., 2009). Furthermore, some H/ACA sRNAs are associated with chromatin and may thus contribute to the regulation of transcription (Schubert et al., 2012). Lastly, H/ACA-like RNAs are critical for trans-splicing in trypanosomes through mediating pseudouridylation of the spliced leader RNA, the substrate for trans-splicing (Barth et al., 2005). Thus, H/ACA sRNAs and their complexes with proteins may have more functions than currently anticipated, and this may also hold true for archaeal H/ACA sRNAs.

## The Assembly Pathway of H/ACA sRNPs

**FIGURE 4:**
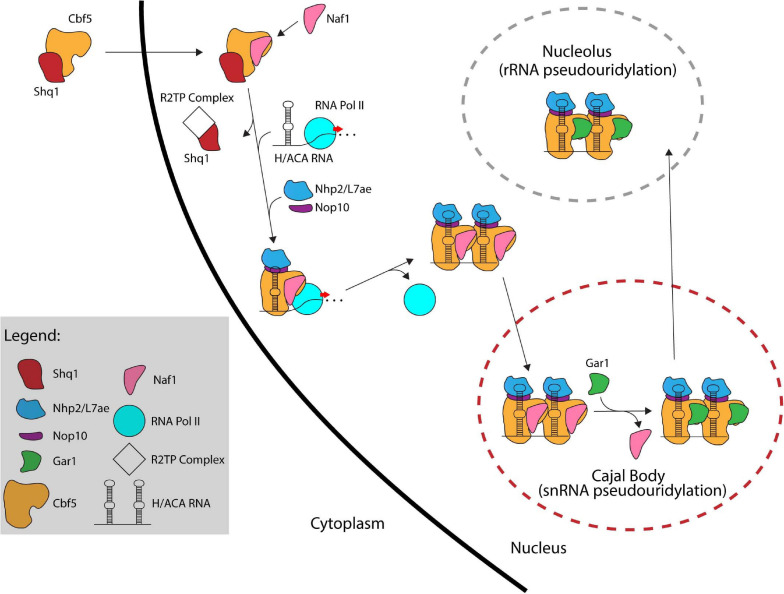
The assembly pathway of eukaryotic H/ACA sRNPs. Several assembly factors like Shq1, Naf1, and the R2TP complex assist in the assembly of eukaryotic H/ACA sRNPs. In contrast, archaeal H/ACA sRNPs are thought to self-assemble without the help of additional factors.

In eukaryotes, the formation of a functional H/ACA sRNP is a complex process that involves several factors working together to assemble and transport the premature H/ACA sRNP particles throughout different compartments of the cell and ultimately to their final location, i.e., the nucleolus or Cajal body (Kiss et al., 2006). In contrast, our current information suggests that archaeal H/ACA sRNPs can self-assemble as none of the additional assembly factors is conserved in archaea. Self-assembly of archaeal H/ACA sRNPs has been successful *in vitro* laying the ground for several biochemical and structural studies (Baker et al., 2005; Charpentier et al., 2005). In contrast, it was much more difficult to reconstitute a yeast H/ACA sRNP in the absence of assembly factors *in vitro* due to the instability of the isolated proteins (Li et al., 2011b; Caton et al., 2018). In the following sections, we will describe the process of H/ACA sRNP biogenesis beginning with the production of nascent Cbf5/dyskerin in the cytoplasm (Figure 4).

Following its translation, Cbf5/dyskerin is quickly bound by Shq1, an essential protein related to Hsp90 cochaperones, that plays a crucial role in early H/ACA sRNP biogenesis by tightly binding the H/ACA sRNA binding interface of Cbf5 through RNA mimicry (Yang et al., 2002; Godin et al., 2009; Walbott et al., 2011). Shq1 binding to Cbf5 ensures that the RNA binding surface of Cbf5 is occupied thereby preventing non-specific RNA binding and aggregation prior to assembly on an H/ACA RNA (Grozdanov et al., 2009; Li et al., 2011a; Caton et al., 2018). Interestingly, mutations in Shq1 can also cause Dyskeratosis congenita (Bizarro and Meier, 2017).

The Cbf5/dyskerin complex with Shq1 is then imported into the nucleus to join the nascent H/ACA sRNA. In *S. cerevisiae*, H/ACA RNAs are typically encoded as single genes (Schattner et al., 2004), whereas H/ACA RNA genes are found within introns of protein coding genes in mammals (Schattner et al., 2006). Through computational and experimental screens, H/ACA snoRNA genes have been identified in several organisms showing a variety of different gene structures such as independent genes, intron-encoded genes, and polycistronic gene clusters (Liang et al., 2004, 2007b; Chen et al., 2008; Wang and Ruvinsky, 2010; Patra Bhattacharya et al., 2016). H/ACA sRNAs are transcribed by RNA Polymerase II followed by processing involving several factors. In yeast, processing of polycistronic H/ACA sRNA is mediated by the endonuclease RNase III (Rnt1) (Chanfreau et al., 1998). Intron-encoded H/ACA sRNAs are typically liberated through splicing and debranching. To mediate further processing in yeast, H/ACA sRNAs are polyadenylated by the poly(A) polymerase Pap1 or the alternative Tfr4, bound by polyA binding protein (Pab2 in fission yeast) and subsequently processed by the nuclear exosome (van Hoof et al., 2000; Grzechnik and Kufel, 2008; Lemay et al., 2010; Berndt et al., 2012). As there are only few pseudouridines in archaeal rRNA and thus only few H/ACA RNAs, the transcription and maturation of archaeal H/ACA RNAs has not been studied in detail, but many archaeal H/ACA RNAs have been identified (Rozhdestvensky et al., 2003; Muller et al., 2007, 2008; Randau, 2015; Toffano-Nioche et al., 2015; Clouet-d’Orval et al., 2018).

After the Cbf5-Shq1 complex enters the nucleus, Cbf5 is bound by the protein Naf1 which contains a Gar1 domain mediating its interaction with Cbf5 (Hoareau-Aveilla et al., 2006; Leulliot et al., 2007). Subsequently, Cbf5 is recruited to the site of H/ACA RNA transcription. In eukaryotes, the recruitment of Cbf5 to the nascent H/ACA RNA is achieved through Naf1-mediated interactions with the C-terminal domain (CTD) of RNA polymerase II (Fatica et al., 2002; Richard et al., 2006). For snoRNAs transcribed from their own promoter in humans, an additional mode of recruitment is suggested that is mediated through TSG1 which is responsible for 5′ hypermethylation of snoRNAs and also interacts with dyskerin (Mouaikel et al., 2002). To enable Cbf5 binding to nascent H/ACA RNA, Shq1 is removed from Cbf5 by the R2TP complex, a multiprotein complex composed of two AAA + ATPases (Rvb1 and Rvb2 in yeast) and two Hsp90 interactors (Pih1 and Tah1 in yeast) that is involved in multiple cellular processes (King et al., 2001; Kakihara and Houry, 2012; Machado-Pinilla et al., 2012). The co-transcriptional assembly of Cbf5 on the H/ACA sRNA is likely protecting the nascent RNA from degradation by exonucleolytic proteins since Cbf5 is necessary for accumulation of all box H/ACA RNAs (Lafontaine et al., 1998; Berndt et al., 2012). Nop10 and Nhp2 are also recruited to the maturing H/ACA sRNP although the timing of their association is not entirely clear. However, the presence of Naf1 prevents Gar1 recruitment and renders the pre-sRNP complex inactive.

Currently, it is not entirely clear whether the Naf1-bound H/ACA pre-sRNPs localize to the Cajal bodies and are then shuttled to the nucleoli, or whether they migrate to the nucleoli directly. In any case, Naf1 is replaced by Gar1 forming the active RNP complexes. Although the process for exchanging these proteins is not fully known, the SMN complex, which like Gar1 is also highly concentrated in Cajal bodies, is implicated in this process supporting the hypothesis that H/ACA sRNPs migrate through the Cajal body (Pellizzoni et al., 2001; Whitehead et al., 2002). Finally, most H/ACA sRNPs are shuttled to the nucleolus to modify ribosomal RNAs while those required for snRNA modification (scaRNAs) remain in the Cajal bodies (Kiss, 2006).

## Discussion

H/ACA sRNPs are versatile ribonucleoprotein machines conserved across both archaea and eukaryotes that play critical roles during ribosome biogenesis through the site-directed formation of pseudouridine modifications in rRNA. In agreement with their conservation, the core structure and functionality of H/ACA sRNPs is the same in archaea and eukaryotes, but multiple adaptations have arisen to further expand the scope of cellular roles of these RNPs such as tRNA modification in archaea as well as modification of several RNAs, rRNA processing, telomerase stabilization, microRNA biogenesis and chromatin regulation in eukaryotes. Notably, some of these additional functions have only emerged recently, and we are still lacking a full understanding of the molecular mechanisms of H/ACA sRNPs in ribosome assembly and beyond. Moreover, H/ACA sRNPs can be utilized as bioengineering devices to site-specifically introduce novel pseudouridines, for example to enable stop codon read-through in yeast (Karijolich and Yu, 2011). As pseudouridines prevent the recognition of mRNA by the immune system and novel mRNA vaccines contain pseudouridines (Kariko et al., 2008; Pardi and Weissman, 2017), the engineering capability of H/ACA sRNPs holds future promising applications beyond the role of H/ACA sRNAs in ribosome formation. Given the current progress in understanding ribosome formation and H/ACA sRNP function, a number of interesting hypotheses are emerging regarding further roles of these ribonucleoproteins. These may hold true in archaea and/or eukaryotes and will likely shape the direction of future research.

Besides stabilizing rRNA through the introduction of pseudouridines, it has been a long-standing speculation that H/ACA sRNPs may also act as rRNA chaperones in both archaea and eukaryotes (Watkins and Bohnsack, 2012; Yip et al., 2013). By base-pairing with rRNA, H/ACA sRNPs may keep certain regions of the rRNA unfolded during the early stages of ribosome assembly thereby preventing premature folding or they may even be able to unfold wrong rRNA folding intermediates. As rRNA folding is a complex and poorly understood process due to the immense size of rRNA, this is an intriguing proposition that will require a concerted approach to be experimentally addressed. In this context, it is interesting to note that eukaryotic H/ACA sRNPs likely rely at least in part on RNA helicases such as Has1 and Rok1 to be removed from rRNA which may contribute to regulating the timing of rRNA folding (Liang and Fournier, 2006; Bohnsack et al., 2008). In contrast, we have no indication to date that helicases fulfill a similar role for H/ACA sRNPs during archaeal ribosome assembly. In addition to rRNA modification and possibly folding, it is noteworthy that one of the most critical functions of a eukaryotic H/ACA sRNA, namely snR30/U17, is to facilitate the processing of pre-rRNA which may also constitute a significant difference between the eukaryotic and archaeal kingdom of life. In all organisms, it will be interesting to understand the coordinated action of H/ACA sRNPs and the other ribosome assembly factors who will interact simultaneously with rRNA early during ribosome formation when the rRNA is still accessible and not yet folded into a compact form. Clearly, many molecular mechanisms and interactions remain to be unraveled regarding ribosome biogenesis in both archaea and eukaryotes.

## Author Contributions

DC and UK devised the conceptual structure of the manuscript. DC wrote the first draft including figures whereas UK refined the text and figures. Both authors contributed to the article and approved the submitted version.

## Conflict of Interest

The authors declare that the research was conducted in the absence of any commercial or financial relationships that could be construed as a potential conflict of interest.
